# LDL-C Concentrations and the 12-SNP LDL-C Score for Polygenic Hypercholesterolaemia in Self-Reported South Asian, Black and Caribbean Participants of the UK Biobank

**DOI:** 10.3389/fgene.2022.845498

**Published:** 2022-03-31

**Authors:** Jasmine Gratton, Chris Finan, Aroon D. Hingorani, Steve E. Humphries, Marta Futema

**Affiliations:** ^1^ Institute of Cardiovascular Science, Faculty of Population Health, University College London, London, United Kingdom; ^2^ UCL British Heart Foundation Research Accelerator, London, United Kingdom; ^3^ Division of Heart and Lungs, Department of Cardiology, University Medical Center Utrecht, Utrecht, Netherlands; ^4^ Cardiology Research Centre, Molecular and Clinical Sciences Research Institute, St George’s, University of London, London, United Kingdom

**Keywords:** FH, ethnicities, UK Biobank, 12-SNP score, LDL-C, polygenic

## Abstract

**Background**: Monogenic familial hypercholesterolaemia (FH) is an autosomal dominant disorder characterised by elevated low-density lipoprotein cholesterol (LDL-C) concentrations due to monogenic mutations in *LDLR*, *APOB*, *PCSK9*, and *APOE*. Some mutation-negative patients have a polygenic cause for elevated LDL-C due to a burden of common LDL-C-raising alleles, as demonstrated in people of White British (WB) ancestry using a 12-single nucleotide polymorphism (SNP) score. This score has yet to be evaluated in people of South Asian (SA), and Black and Caribbean (BC) ethnicities.

**Objectives**: 1) Compare the LDL-C and 12-SNP score distributions across the three major ethnic groups in the United Kingdom: WB, SA, and BC individuals; 2) compare the association of the 12-SNP score with LDL-C in these groups; 3) evaluate ethnicity-specific and WB 12-SNP score decile cut-off values, applied to SA and BC ethnicities, in predicting LDL-C concentrations and hypercholesterolaemia (LDL-C>4.9 mmol/L).

**Methods**: The United Kingdom Biobank cohort was used to analyse the LDL-C (adjusted for statin use) and 12-SNP score distributions in self-reported WB (*n* = 353,166), SA (*n* = 7,016), and BC (*n* = 7,082) participants. To evaluate WB and ethnicity-specific 12-SNP score deciles, the total dataset was split 50:50 into a training and testing dataset. Regression analyses (logistic and linear) were used to analyse hypercholesterolaemia (LDL-C>4.9 mmol/L) and LDL-C.

**Findings**: The mean (±SD) measured LDL-C differed significantly between the ethnic groups and was highest in WB [3.73 (±0.85) mmol/L], followed by SA [3.57 (±0.86) mmol/L, *p* < 2.2 × 10^−16^], and BC [3.42 (±0.90) mmol/L] participants (*p* < 2.2 × 10^−16^). There were significant differences in the mean (±SD) 12-SNP score between WB [0.90 (±0.23)] and BC [0.72 (±0.25), *p* < 2.2 × 10^−16^], and WB and SA participants [0.86 (±0.19), *p* < 2.2 × 10^−16^]. In all three ethnic groups the 12-SNP score was associated with measured LDL-C [*R* ^2^ (95% CI): WB = 0.067 (0.065–0.069), BC = 0.080 (0.063–0.097), SA = 0.027 (0.016–0.038)]. The odds ratio and the area under the curve for hypercholesterolaemia were not statistically different when applying ethnicity-specific or WB deciles in all ethnic groups.

**Interpretation**: We provide information on the differences in LDL-C and the 12-SNP score distributions in self-reported WB, SA, and BC individuals of the United Kingdom Biobank. We report the association between the 12-SNP score and LDL-C in these ethnic groups. We evaluate the performance of ethnicity-specific and WB 12-SNP score deciles in predicting LDL-C and hypercholesterolaemia.

## Introduction

Pathogenic variants in *LDLR, APOB, PCSK9*, and *APOE* are known to cause autosomal dominant familial hypercholesterolaemia (FH), but such mutations are found in only ∼40% of individuals with the clinical diagnosis of FH (Taylor et al., 2010; Motazacker et al., 2012; Futema et al., 2013; Nordestgaard et al., 2013; Mariano et al., 2020). In 2013, Talmud et al. (2013) reported that a high burden of common variants identified from genome-wide association studies (GWAS) was associated with LDL-C concentrations as high or higher than those found in individuals with a FH mutation, suggesting a polygenic cause in mutation negative patients with a biochemical FH phenotype. A score based on 12-single nucleotide polymorphisms (SNP) was developed to suggest a polygenic cause of high LDL-C concentrations for these FH mutation-negative individuals (Talmud et al., 2013). The validity of this score has been confirmed in samples of no-mutation FH adults and children from more than 8 countries with White European populations (Futema et al., 2018). Other studies applied the score to define a polygenic cause of high LDL-C in patients with hypercholesterolaemia (Amor-Salamanca et al., 2017; Saadatagah et al., 2021). Two of the current NHS Genomic Laboratory Hubs (GLHs) (South West and North East) are including the 12-SNP score within their diagnostic pipelines (George et al., 2021), demonstrating feasibility of implementation. Clinicians receiving these reports are strongly supportive of the roll out to all GLHs and find them helpful for patient management because of the benefit of being able to offer a genetic explanation for mutation negative patients with a biochemical FH phenotype, which may motivate adherence to lifestyle interventions and cholesterol-lowering therapy (Kullo et al., 2016). However, the major limitation of the 12-SNP score is that it has not yet been evaluated in two other major ethnic groups in the United Kingdom [South Asian (SA), and Black and Caribbean (BC) individuals], which has led to uncertainty in reporting. In this study, using the United Kingdom Biobank data we analysed the LDL-C and 12-SNP score distributions among self-reported WB, SA, and BC participants; and tested the association between the 12-SNP score and LDL-C concentrations in the three groups. We also performed an out-of-sample validation to compare the use of WB versus ethnicity-specific 12-SNP score deciles applied to SA and BC participants.

## Materials and Methods

The analysis was performed in individuals of self-reported White (*n* = 353,166), South Asian (SA) (*n* = 7,016), and Black and Caribbean (BC) (*n* = 7,082) ethnicities of the United Kingdom Biobank (project ID 40721). Up to third degree relatives were excluded from the dataset. The following variables had missing data which was singly imputed using predictive mean matching (PMM) with the R package “mice” (van Buuren and Groothuis-Oudshoorn 2011): LDL-C, body mass index (BMI), total cholesterol, high-density lipoprotein cholesterol (HDL-C), triglycerides, and smoking. The data analysis was performed in R version 4.0.2. Measured LDL-C concentrations (UKB data-field 30,690) were adjusted for self-reported statin use by multiplying their measured LDL-C concentrations by the correction coefficient 1.43, as used by Trinder et al. (2020). The weighted 12-SNP scores were calculated for each participant using the previously published SNPs and effect sizes from people of European ancestry (Supplementary Table S1) (Talmud et al., 2013). The two *APOE* variants were calculated in an isoform-specific manner as detailed in Supplementary Table S1 (Bennet et al., 2007). The 10 other variants and their effect sizes (also known as the beta value, which is the incremental increase or decrease in LDL-C concentration per effect allele), originally obtained from the Global Lipids Genetics Consortium (GLGC) GWAS (Teslovich et al., 2010), were calculated by first converting the negative effect sizes to positive ones and inverting the effect and non-effect alleles; and subsequently, the number of alleles multiplied by their positive effect sizes were summed for each individual. The scores obtained from the *APOE* isoforms and the GLGC variants were then added together to obtain the final 12-SNP score for each participant.

The UK Biobank dataset was split 50:50 into a training and a testing dataset. Ethnicity-specific 12-SNP score decile cut-off values were obtained for each self-reported ethnic group using the training data. These cut-points were applied to the test data in an ethnicity-specific manner. The WB 12-SNP score decile cut-off values obtained from the training data were also applied to the test data in SA and BC participants to compare the performance of WB and ethnicity-specific deciles. The test data was used for the analyses performed in this study. Regression analyses (linear and logistic) were used to predict LDL-C concentration and hypercholesterolaemia (LDL-C >4.9 mmol/L, one of the FH diagnostic criteria included in the Simon Broome criteria) (Civeira et al., 2004). Individuals were also grouped into low (deciles 1–3), intermediate (deciles 4–5), and high (deciles 6–10) polygenic hypercholesterolaemia groups based on their 12-SNP score. The odds ratio (OR) for hypercholesterolaemia was obtained by setting the intermediate polygenic hypercholesterolaemia group as the reference.

The analysis was also performed in principal component analysis (PCA)-verified White European, Black, and South Asian ancestry groups as defined by Giannakopoulou et al. (2021)(Supplementary Table S2).

## Results

The study participant characteristics were significantly different for self-reported White (WB), South Asian (SA) and Black and Caribbean (BC) ethnic groups (Table 1). WB participants were older (median age of 58 [interquartile range (IQR): 51–63)] and exhibited the highest concentrations of adjusted LDL-C [3.73 mmol/L (standard deviation (SD): 0.85], total cholesterol [5.71 mmol/L (SD: 1.14)], high-density lipoprotein cholesterol (HDL-C) [1.46 mmol/L (SD: 0.41)] and non-HDL-C [4.26 mmol/L (SD: 1.07)] compared to SA and BC individuals. A higher proportion of males was present in the SA ethnic group (54.3%) compared to the WB (46.2%) and BC (43.3%) ethnic groups. 80.5% of SA and 72.7% of BC participants were self-reported non-smokers, while 56.4% WB participants self-reported as non-smoking. Mean body mass index (BMI) was highest in BC participants [29.46 kg/m^2^ (SD: 5.36)], and there was no significant difference in mean BMI between WB and SA participants (*p*-value = 0.11) (Table 1). Triglyceride levels were highest for SA participants [1.67 mmol/L (IQR: 1.18–2.40)], as well as the use of statins (21%), and the prevalence (6.8%) and incidence (7%) of coronary heart disease (CHD).

**TABLE 1:** **S**tudy participant characteristics stratified by self-reported ethnicity. For continuous variables, *p*-values from the Welch t-statistic tests are reported; while for categorical and binary variables, *p*-values from Pearson’s Chi-squared tests are reported. The data presented here is for the training and the test dataset combined. CHD, coronary heart disease; HDL-C, high-density lipoprotein cholesterol; IQR, interquartile range; LDL-C, low-density lipoprotein cholesterol; SD , standard deviation; SNP, single nucleotide polymorphism.

	White	Black/Caribbean	South Asian	P-Value: White-Black/Caribbean	P-Value: White-South Asian
n	353,166	7,082	7,016		
Age (median [IQR])	58 [51, 63]	50 [45, 58]	53 [46, 60]	<2.2 × 10^−16^	<2.2 × 10^−16^
Sex (male) (%)	163,063 (46.2)	3,070 (43.3)	3,807 (54.3)	2.5 × 10^−6^	<2.2 × 10^−16^
Body mass index, kg/m2 [mean (SD)]	27.39 (4.76)	29.46 (5.36)	27.31 (4.47)	<2.2 × 10^−16^	0.11
Smoking status (%)				<2.2 × 10^−16^	<2.2 × 10^−16^
Non-smoker	199,045 (56.4)	5,147 (72.7)	5,646 (80.5)		
Former smoker	129,004 (36.5)	1,357 (19.2)	961 (13.7)		
Light smoker (<10 cigarettes/day)	4,932 (1.4)	240 (3.4)	139 (2.0)		
Moderate smoker (10–19 cigarettes/day)	10,698 (3.0)	257 (3.6)	186 (2.7)		
Heavy Smoker (>20 cigarettes/day)	9,487 (2.7)	81 (1.1)	84 (1.2)		
Blood biomarkers					
LDL-C (unadjusted), mmol/L [mean (SD)]	3.57 (0.87)	3.27 (0.84)	3.33 (0.85)	<2.2 × 10^−16^	<2.2 × 10^−16^
LDL-C (adjusted for statin users), mmol/L [mean (SD)]	3.73 (0.85)	3.42 (0.90)	3.57 (0.86)	<2.2 × 10^−16^	<2.2 × 10^−16^
Total cholesterol, mmol/L [mean (SD)]	5.71 (1.14)	5.26 (1.10)	5.29 (1.11)	<2.2 × 10^−16^	<2.2 × 10^−16^
Triglycerides, mmol/L [median (IQR)[	1.49 [1.05, 2.16]	1.05 [0.77, 1.50]	1.67 [1.18, 2.40]	<2.2 × 10^−16^	<2.2 × 10^−16^
HDL-C, mmol/L [mean (SD)]	1.46 (0.41)	1.45 (0.41)	1.27 (0.36)	2.4 × 10^−4^	<2.2 × 10^−16^
Non-HDL-C, mmol/L [mean (SD)]	4.26 (1.07)	3.83 (1.01)	4.03 (1.05)	<2.2 × 10^−16^	<2.2 × 10^−16^
Apolipoprotein A, g/L [mean (SD)]	1.54 (0.27)	1.49 (0.26)	1.40 (0.24)	<2.2 × 10^−16^	<2.2 × 10^−16^
Apolipoprotein B, g/L [mean (SD)]	1.03 (0.24)	0.97 (0.24)	1.00 (0.23)	<2.2 × 10^−16^	<2.2 × 10^−16^
Statin use (%)	47,427 (13.4)	873 (12.3)	1,475 (21.0)	7.0 × 10^−3^	<2.2 × 10^−16^
12-SNP score [mean (SD)]	0.90 (0.23)	0.72 (0.25)	0.86 (0.19)	<2.2 × 10^−16^	<2.2 × 10^−16^
Prevalent CHD (%)	11,743 (3.3)	115 (1.6)	479 (6.8)	2.5 × 10^−15^	<2.2 × 10^−16^
Incident CHD (%)	14,471 (4.1)	200 (2.8)	491 (7.0)	9.4 × 10^−8^	<2.2 × 10^−16^

**FIGURE 1:**
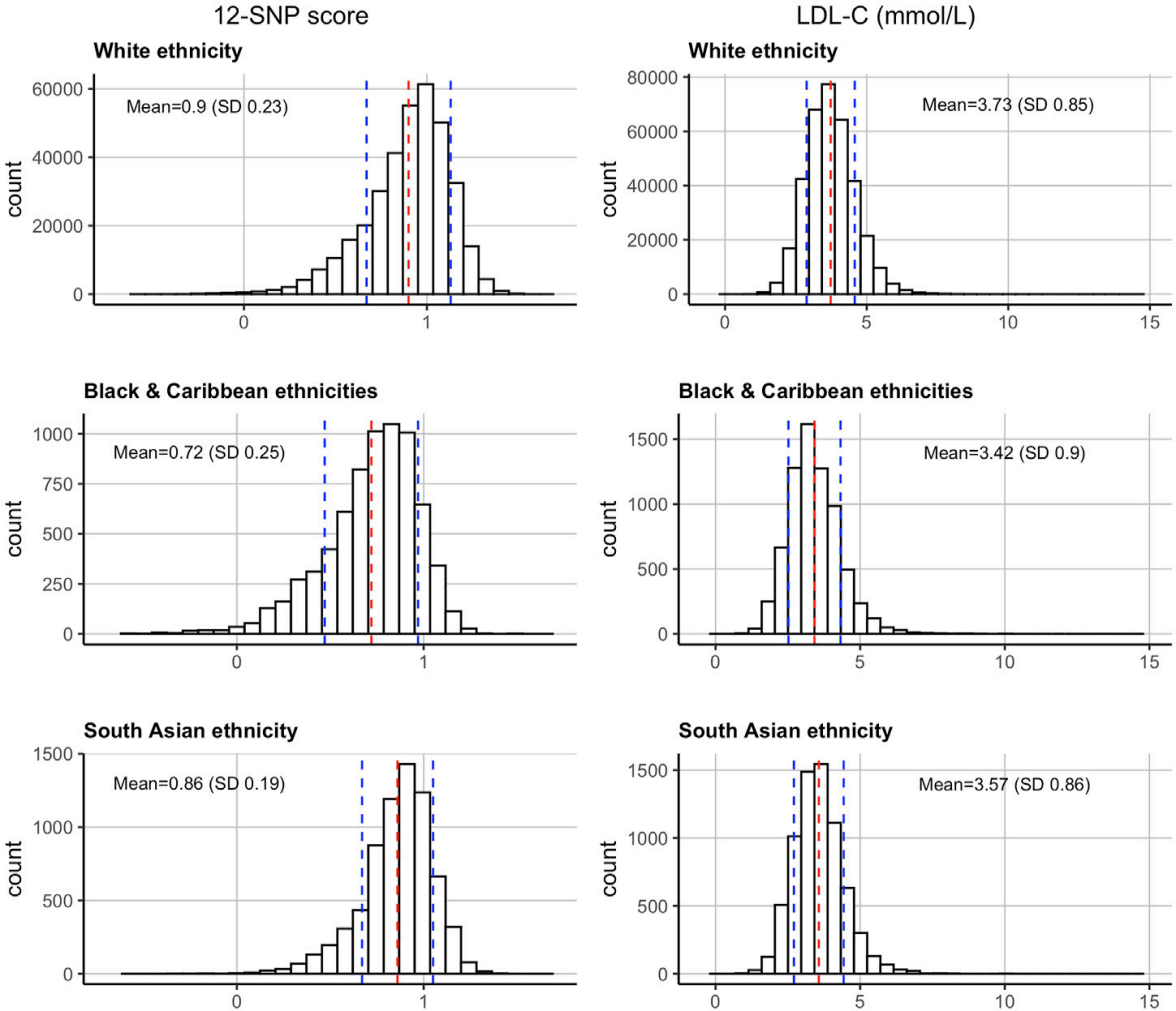
Distributions of the 12-SNP score and LDL-C concentrations in the United Kingdom Biobank in self-reported White, Black & Caribbean, and South Asian ethnicities. The mean and SD are depicted by dotted red and blue lines respectively. LDL-C, low-density lipoprotein cholesterol; SD, standard deviation.

When compared between the three ethnic groups, the measured mean (SD) LDL-C concentrations adjusted for statin use was highest in WB participants [3.73 (0.85) mmol/L], followed by SA [3.57 (0.86) mmol/L], and BC [3.42 (0.90) mmol/L] participants (Figure 1). These group differences were statistically significant (*p*-value <2.2 × 10^−16^) (Table 1).

The 12-SNP score followed the same pattern, with the mean (SD) score being the highest in WB participants [0.90 (0.23)], followed by SA [0.86 (0.19)], and BC [0.72 (0.25)] participants (Table 1 and Figure 1). These group differences were also statistically significant (*p*-value <2.2 × 10^−16^). The ethnicity-specific 12-SNP score decile cut-off values were therefore different between WB, BC, and SA participants (Table 2).

**TABLE 2:** Outcome data according to self-reported ethnicity-specific 12-SNP score deciles. The results presented here are using the test data. The training data was used to obtain the 12-SNP score decile cut-off values for each ethnic group studied. LDL-C, low-density lipoprotein cholesterol; SD, standard deviation; SNP, single nucleotide polymorphism.

Self-reported ethnicity	12-SNP score decile	Minimum decile value	Maximum decile value	Number of individuals	Mean 12-SNP score	SD 12-SNP score	Mean LDL-C (mmol/L)	SD LDL-C (mmol/L)	Number of individuals with LDL-C >4.9 mmol/L	Percent (%) of individuals with LDL-C >4.9 mmol/L
White	1	−0.54	0.58	17,731	0.42	0.15	3.27	0.78	505	2.8
White	2	0.58	0.73	17,846	0.66	0.04	3.49	0.77	746	4.2
White	3	0.73	0.82	17,457	0.77	0.03	3.61	0.79	1,018	5.8
White	4	0.82	0.88	17,915	0.85	0.02	3.69	0.82	1,320	7.4
White	5	0.88	0.93	17,707	0.91	0.02	3.74	0.82	1,422	8
White	6	0.93	0.98	17,371	0.96	0.01	3.8	0.83	1,633	9.4
White	7	0.98	1.03	17,395	1.01	0.01	3.83	0.85	1758	10.1
White	8	1.03	1.08	17,551	1.06	0.02	3.89	0.84	1983	11.3
White	9	1.08	1.16	17,920	1.12	0.02	3.94	0.86	2,230	12.4
White	10	1.16	1.56	17,150	1.22	0.06	4.03	0.88	2,682	15.6
Black & Caribbean	1	−0.59	0.38	383	0.2	0.17	2.92	0.79	9	2.3
Black & Caribbean	2	0.38	0.53	346	0.46	0.04	3.14	0.77	6	1.7
Black & Caribbean	3	0.53	0.63	340	0.58	0.03	3.25	0.87	9	2.6
Black & Caribbean	4	0.63	0.7	336	0.67	0.02	3.44	0.88	22	6.5
Black & Caribbean	5	0.7	0.76	315	0.73	0.02	3.42	0.85	13	4.1
Black & Caribbean	6	0.76	0.81	336	0.78	0.02	3.42	0.9	21	6.2
Black & Caribbean	7	0.81	0.87	381	0.84	0.02	3.52	0.81	20	5.2
Black & Caribbean	8	0.87	0.93	378	0.9	0.02	3.62	0.88	32	8.5
Black & Caribbean	9	0.93	1	352	0.96	0.02	3.75	1.01	31	8.8
Black & Caribbean	10	1	1.47	325	1.07	0.06	3.76	0.95	40	12.3
South Asian	1	−0.17	0.61	342	0.46	0.12	3.29	0.8	11	3.2
South Asian	2	0.61	0.73	356	0.67	0.04	3.46	0.82	20	5.6
South Asian	3	0.73	0.79	377	0.76	0.02	3.53	0.8	15	4
South Asian	4	0.79	0.84	311	0.82	0.01	3.5	0.84	19	6.1
South Asian	5	0.84	0.88	361	0.86	0.01	3.58	0.8	19	5.3
South Asian	6	0.88	0.93	368	0.91	0.01	3.62	0.84	24	6.5
South Asian	7	0.93	0.96	323	0.95	0.01	3.57	0.81	18	5.6
South Asian	8	0.96	1.01	362	0.99	0.01	3.73	1	40	11
South Asian	9	1.01	1.08	377	1.04	0.02	3.68	0.92	32	8.5
South Asian	10	1.08	1.38	279	1.15	0.06	3.81	0.92	27	9.7

**FIGURE 2:**
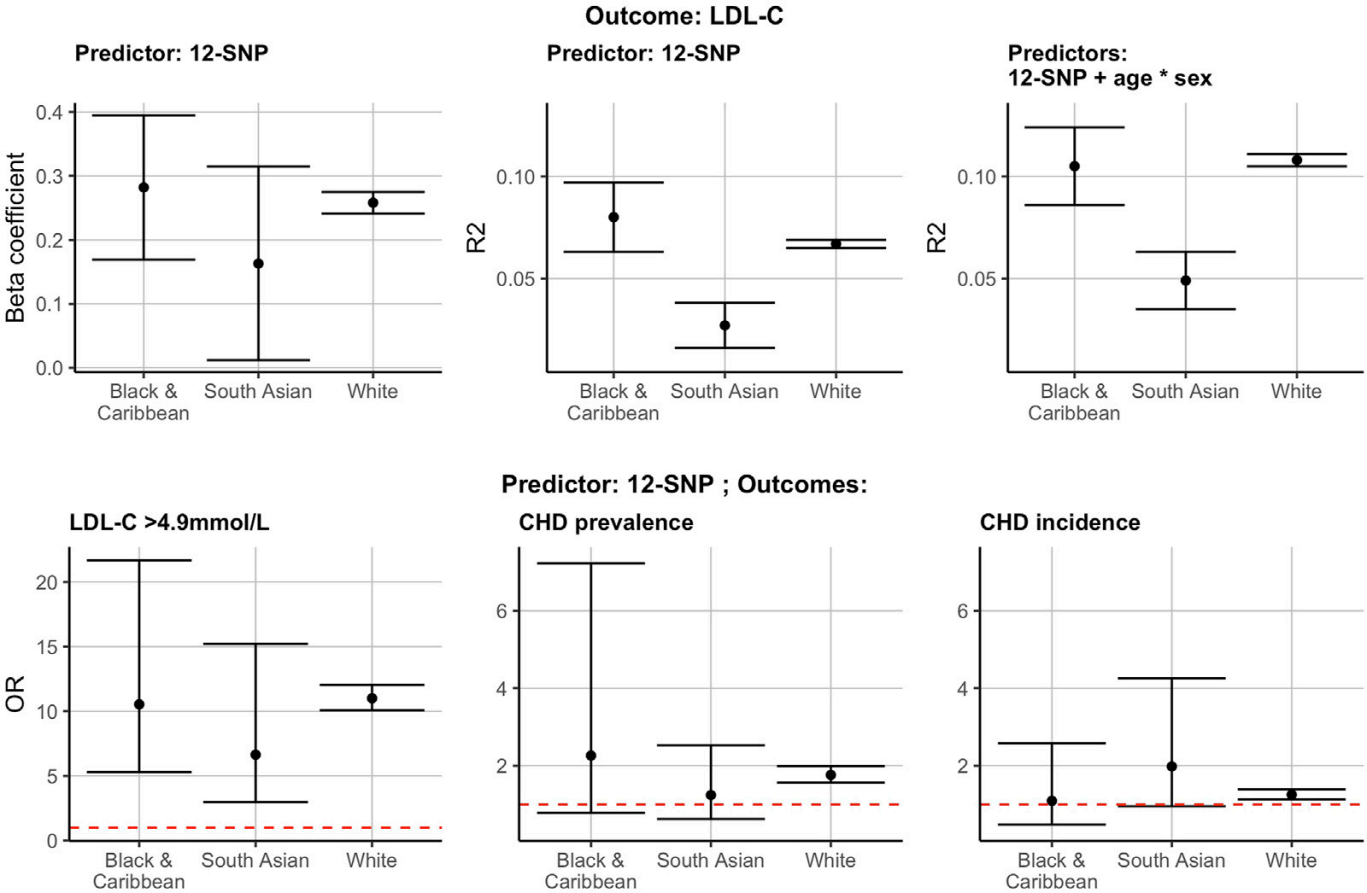
The 12-SNP score in the prediction of LDL-C, hypercholesterolaemia (LDL-C >4.9 mmol/L) and CHD prevalence and incidence in self-reported BC, SA and WB ethnicities. The results presented here are using the test data. * denotes the interaction term in the regression analysis. The red dotted horizontal lines represent an OR of 1. CHD, coronary heart disease; LDL-C, low-density lipoprotein cholesterol; R2, correlation coefficient (*R* ^2^); OR, odds ratio; BC, Black & Caribbean; SA, South Asian; WB, White British.

In all three ethnic groups studied, the 12-SNP score was correlated with LDL-C concentrations: the *R* ^2^ was equal to 0.067 [95% confidence intervals (CI): 0.065–0.069] in WB, 0.080 (95% CI: 0.063–0.097) in BC, and 0.027 (95% CI: 0.016–0.038) in SA individuals (Supplementary Table S3 and Figure 2). The beta coefficients indicating the increase in LDL-C per unit increase of the score were equal to 0.258 (95% CI: 0.241–0.275) for WB, 0.282 (95% CI: 0.169–0.395) for BC, and 0.163 (95% CI: 0.012–0.315) for SA participants (Supplementary Table S3 and Figure 2). The 12-SNP score had an OR of 11.01 (95% CI: 10.08–12.04) in WB, 10.54 (95% CI: 5.29–21.67) in BC, and 6.64 (95% CI: 2.98–15.22) in SA individuals in predicting hypercholesterolaemia (LDL-C >4.9 mmol/L) (Supplementary Table S3 and Figure 2).

The 12-SNP score was also associated with an increased OR for CHD prevalence and incidence in self-reported WB individuals [CHD prevalence: 1.76 (95% CI: 1.56–1.99); CHD incidence: 1.25 (95% CI: 1.13–1.39)] (Figure 2 and Supplementary Table S3). These observations were also present in self-reported SA and BC participants, but the CI were wide and overlapping with 1 (Figure 2 and Supplementary Table S3).

**FIGURE 3:**
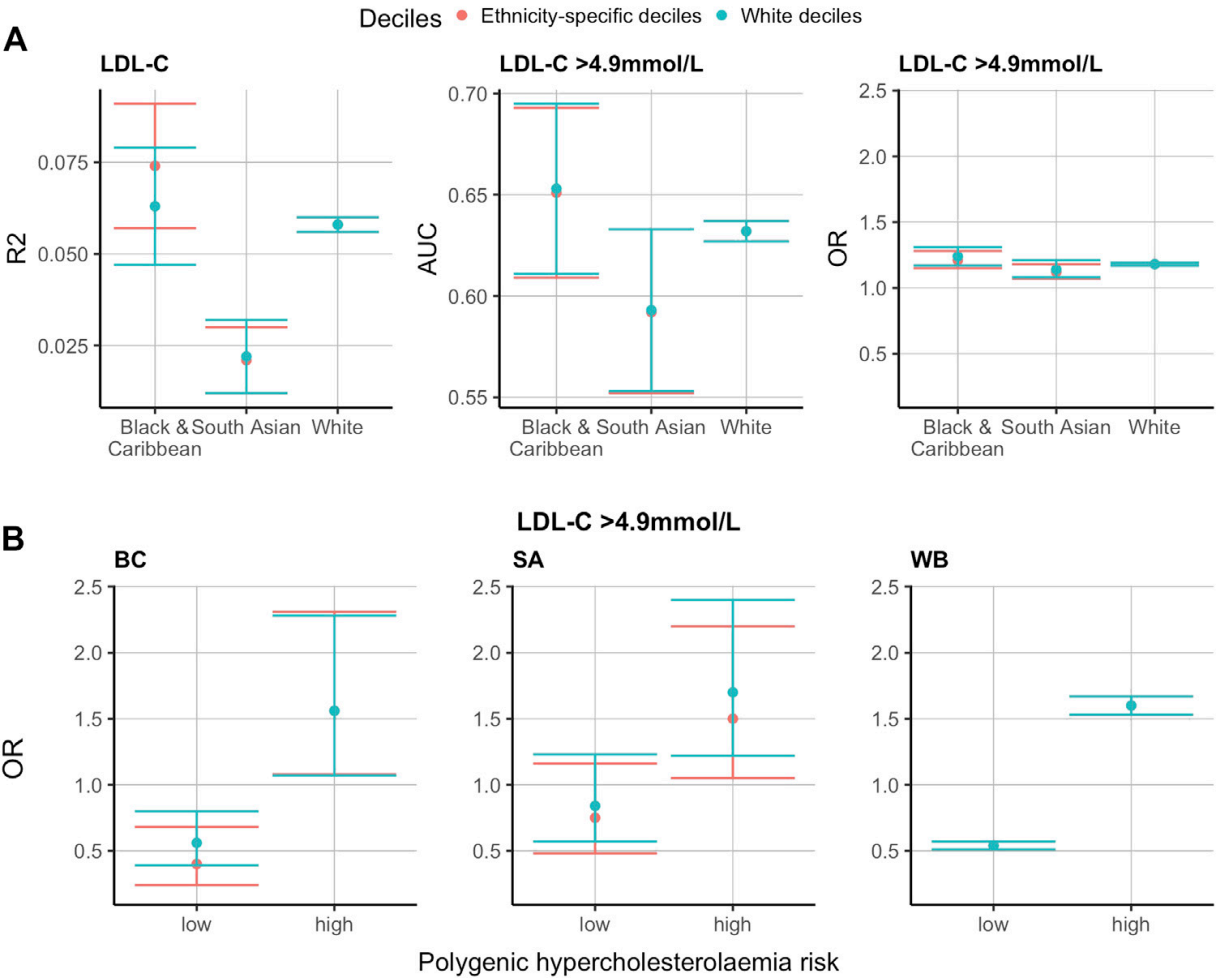
Ethnicity-specific versus WB 12-SNP score decile cut-off values in the prediction of LDL-C and hypercholesterolaemia (LDL-C >4.9 mmol/L) in self-reported BC, SA and WB ethnicities. The results presented here are using the test data. The training data was used to obtain the 12-SNP score decile cut-off values for each ethnic group studied. The red and blue plots refer to ethnicity-specific and White deciles respectively. **(A).** *R* ^2^, AUC and OR for the prediction of LDL-C and hypercholesterolaemia (LDL-C >4.9 mmol/L) using ethnicity-specific versus WB 12-SNP score decile cut-off values. **(B)**. Polygenic hypercholesterolaemia risk using ethnicity-specific versus WB 12-SNP score deciles cut-off values for the prediction of hypercholesterolaemia (LDL-C >4.9 mmol/L). Low polygenic hypercholesterolaemia risk refers to the 12-SNP score deciles 1-3, intermediate polygenic hypercholesterolaemia risk represent deciles 4-5, and high polygenic hypercholesterolaemia risk are for deciles 6–10. Intermediate polygenic hypercholesterolaemia risk is the reference group equal to an OR of 1. LDL-C, low-density lipoprotein cholesterol; R2, correlation coefficient (*R* ^2^); OR, odds ratio; AUC, area under the curve; BC, Black & Caribbean; SA, South Asian; WB, White British.

Considering that the LDL-C and 12-SNP score distributions were significantly different between the three ethnic groups studied, we hypothesised that applying ethnicity-specific 12-SNP score deciles might be more accurate in predicting mean LDL-C concentrations and hypercholesterolaemia (defined as LDL-C >4.9 mmol/L by the Simon Broome criteria). To test this, we split the data 50:50 into a training and a testing dataset and applied the WB and ethnicity-specific decile cut-off values derived from the training data to the test data (Supplementary Table S4). We then tested the association between the deciles and the mean adjusted LDL-C concentrations in WB, SA, and BC participants. In all ethnic groups studied, the mean adjusted LDL-C concentration had a positive *R* ^2^ value with the ethnicity-specific 12-SNP score deciles: 0.058 (95% CI: 0.056–0.060) for WB, 0.063 (95% CI: 0.047–0.079) for BC, and 0.022 (95% CI: 0.012–0.032) for SA participants (Supplementary Table S3 and Figure 3). The *R* ^2^ obtained using the WB 12-SNP score deciles had overlapping confidence intervals with the ethnicity-specific deciles: 0.074 (95% CI: 0.057–0.091) for BC, and 0.021 (95% CI: 0.012–0.030) for SA participants (Supplementary Table S3 and Figure 3). For hypercholesterolaemia (LDL-C >4.9 mmol/L), the area under the curve (AUC) was identical when applying the ethnicity-specific deciles or when applying the WB deciles: 0.63 (95% CI: 0.63–0.64) for WB, 0.65 (95% CI: 0.61–0.69) for BC, and 0.59 (95% CI: 0.55–0.63) for SA individuals (Supplementary Table S3 and Figure 3).

When grouping individuals into low (deciles 1-3 of the 12-SNP score), intermediate (deciles 4–5), and high (deciles 6–10) polygenic hypercholesterolaemia categories and using the intermediate group as the reference category, the OR was lowest in the low polygenic hypercholesterolaemia category and highest in the high polygenic hypercholesterolaemia category (Supplementary Table S3 and Figure 3). The values obtained were similar in the three ethnic groups studied: for low polygenic hypercholesterolaemia, the OR (95% CI) was 0.54 (0.51–0.57) for WB individuals, 0.40 (0.24–0.68) for BC individuals, and 0.75 (0.48–1.16) for SA individuals; and for the high polygenic hypercholesterolaemia group, the OR (95% CI) was 1.60 (1.53–1.67) for WB individuals, 1.56 (1.08–2.31) for BC individuals, and 1.50 (1.05–2.20) for SA individuals (Supplementary Table S3 and Figure 3). The OR were similar when applying the ethnicity-specific 12-SNP score decile cut-off values and the WB decile cut-off values: when applying the WB deciles, the OR (95% CI) for BC individuals was equal to 0.56 (0.39–0.80) for the low polygenic hypercholesterolaemia category, and to 1.56 (1.07–2.28) for the high polygenic hypercholesterolaemia category; and for SA individuals, the OR (95% CI) was equal to 0.84 (0.57–1.23) for the low polygenic hypercholesterolaemia category, and to 1.70 (1.22–2.40) for the high polygenic hypercholesterolaemia category (Supplementary Table S3 and Figure 3).

The results for PCA-verified White, Black, and South Asian ancestries were similar to the results obtained for self-reported WB, BC, and SA individuals (Supplementary Table S2).

## Discussion

In this study, we analysed the adjusted LDL-C concentrations and 12-SNP LDL-C score distributions for three major ethnic groups of the UK Biobank and the UK: White (WB), Black and Caribbean (BC), and South Asian (SA). The rationale for using self-reported ethnicity in our analysis instead of PCA-verified ancestry is to mimic a typical clinical scenario where the 12-SNP score would be applied. However, we also performed the analysis in PCA-verified White, Black, and South Asian ancestries and obtained similar results (Supplementary Table S2). We observed that the adjusted LDL-C mean values were significantly different between WB and BC, and between WB and SA ethnicities. This likely reflects both the differences in genetic and environmental factors that influence LDL-C concentrations. One explanation is that several of the participant characteristics that are known to influence LDL-C concentrations (age, sex, and statin use) were significantly different between WB and SA participants, and WB and BC participants.

The 12-SNP score distributions were also significantly different between WB and SA, and between WB and BC participants. These differences in the score distributions are likely to be due to the differences in the minor allele frequencies (MAFs) of the 12 variants included in the score between these ethnic groups (Supplementary Table S1). All 12 SNPs showed significant differences in MAF between the three ancestry groups, with for example rs1800562 in the *HFE* gene being tenfold more frequent in WB compared to SA and BC individuals (Supplementary Table S1). Since the 12-SNP score variants and weights were derived in a White European population (Teslovich et al., 2010), to further improve the identification of polygenic causes of hypercholesterolaemia among SA and BC patients, and once GWAS increase in diversity and size, the SNPs selected in the score should be re-evaluated to reflect these genetic differences. In a recent report from GLGC, a large-scale multi-ancestry GWAS meta-analysis of lipid levels demonstrated that an LDL-C polygenic risk score (PRS) developed from a diverse population performed better than an ancestry-specific PRS (Graham et al., 2021).

In all three ethnic groups, the 12-SNP score was correlated with LDL-C concentrations, with the highest *R* ^2^ obtained for BC, followed by WB, and finally SA individuals, although the confidence intervals were overlapping for BC and WB individuals suggesting that it performed equally well in BC and WB individuals. This also suggests that the 12-SNP score is better suited for individuals of self-reported BC ethnicity compared to individuals of self-reported SA ethnicity, although the variance in LDL-C explained by the 12-SNP score remains low in all ethnic groups (highest estimate obtained: *R* ^2^ 8%). Including more variants in the score might improve the LDL-C variance observed. A recent study by Wu et al. (2021) showed that a polygenic score for LDL-C containing 8,367 variants had a *R* ^2^ of 0.215 (95% CI: 0.207–0.222) in WB individuals and a *R* ^2^ of 0.139 (95% CI: 0.125–0.154) in SA individuals of the UK Biobank, although such a large score would be currently less feasible to implement in the clinic. And in the recent GLGC study based on the largest genotype and lipids data to date, several LDL-C PRS were tested and the polygenic predictability of LDL-C adjusted for covariates (adjusted *R* ^2^) varied between 0.10 and 0.16 across the five PCA-verified ancestry groups studied (Graham et al., 2021).

The WB 12-SNP score decile cut-off values in this study were almost identical (up until the second decimal place) to the published values derived from the UK Whitehall II study (Talmud et al., 2013). This supports the robustness of the 12-SNP decile cut-off values for WB individuals. Since the 12-SNP score is already being reported by several diagnostic labs across the UK, we provide information on how the ethnicity-specific deciles and the WB deciles of the 12-SNP score applied to these ethnic groups perform.

The 12-SNP score decile cut-off values obtained for WB, SA and BC were different. Considering that both the 12-SNP score and LDL-C distributions were significantly different between the ethnic groups studied, we hypothesised that applying ethnicity-specific decile cut-off values might be more appropriate than using WB 12-SNP score decile cut-off values. The out-of-sample validation results suggest that the WB 12-SNP score deciles applied to SA and BC participants, and the ethnicity-specific 12-SNP score deciles perform similarly in SA and BC ethnicities when predicting both the adjusted LDL-C concentrations or hypercholesterolaemia (defined as LDL-C >4.9 mmol/L by the FH Simon Broome diagnostic criteria).

Diagnostic labs in the UK are grouping individuals into low (deciles 1–3), intermediate (deciles 4–5) and high (deciles 6–10) polygenic hypercholesterolaemia score categories in order to simplify the classification of polygenic hypercholesterolaemia (George et al., 2021). This also ensures that the discrepancies in the percent of individuals with LDL-C >4.9 mmol/L in each decile are smoothed for the BC and SA ethnic groups. The grouping into low, intermediate, and high polygenic hypercholesterolaemia categories shows an increase in the odds ratio for hypercholesterolaemia from one category to another in all self-reported ethnic groups when applying the WB 12-SNP score deciles and the ethnicity-specific 12-SNP score deciles. Overall, the odds ratios for hypercholesterolaemia were similar for all ethnic groups (or had overlapping confidence intervals), implying that the ethnic differences in the 12-SNP score might not be as large as the ones reported by other polygenic score studies (Martin et al., 2019), most likely due to the low number of variants in the score, the small sample size of SA and BC individuals, and because the self-reported ethnicities in this study were not PCA verified. Indeed, the 95% confidence intervals obtained using the ethnicity-specific deciles overlapped between the low and high polygenic hypercholesterolaemia categories in SA individuals, and this was also the case (by a value of 0.01) when applying the WB decile cut-off values. However, this was not observed when using PCA-verified SA ancestry as a study cohort, which might be because the sample size was bigger (8279 PCA-verified individuals of SA ancestry versus 7,016 participants of self-reported SA ethnicity), leading to narrower CI and more accurate estimates (Supplementary Tables S2, S3).

A limitation of this study is that monogenic FH cases in the cohort were not identified because the exome data was not readily available for all participants. Based on the estimated prevalence of 1:250 monogenic FH cases in the general population (Akioyamen et al., 2017), we expect roughly 1,469 (0.4%) participants in our cohort to have a monogenic cause for their hypercholesterolaemia, as opposed to a polygenic cause. However, it is unlikely that this relatively small number of monogenic FH cases would significantly influence our results. Overall, environmental variables are likely to have the biggest influence on LDL-C concentrations in all ethnic groups. Another limitation is that the sample size for BC (*n* = 7,082) and SA (*n* = 7,016) individuals were small compared to WB (*n* = 353,166) individuals, resulting in less precise estimates than for WB individuals, and potentially also explaining why the ethnicity-specific deciles were not significantly more accurate in predicting LDL-C concentrations and hypercholesterolaemia. Ideally this analysis would be replicated in an external and larger dataset to get more accurate decile cut-off values and estimates.

The 12-SNP score is currently being used in clinical practice in two GLHs in the UK. The score is being administered to FH mutation-negative patients with hypercholesterolaemia in order to provide them with an explanation for their hypercholesterolaemia (i.e., a polygenic cause or not). The aim of this study was not to evaluate the utility of the score in clinical practice, but to provide information on how this score, which was developed in individuals of White ancestry, performs in individuals of self-reported SA and BC ethnicities. We found that this 12-SNP score translated better to individuals of self-reported BC ethnicity than to individuals of SA ethnicity. We also found that ethnicity-specific deciles of the score performed as well as WB deciles in these two ethnic groups.

Overall, LDL-C concentrations are likely to be heavily influenced by environmental factors, and to varying levels in different ethnic groups. Regardless of cause, the key is to focus on lowering LDL-C concentrations in individuals with hypercholesterolaemia through the means of cholesterol-lowering therapy and by advocating a healthy diet and lifestyle. More research is also needed to fully assess the benefits and risks of returning polygenic information to patients (Adeyemo et al., 2021).

## Data Availability

The data analyzed in this study is subject to the following licenses/restrictions: UK Biobank application ID 40721. Requests to access these datasets should be directed to, https://www.ukbiobank.ac.uk/enable-your-research/apply-for-access.
