# Supplementary material for: LDL-C Concentrations and the 12-SNP LDL-C Score for Polygenic Hypercholesterolaemia in Self-Reported South Asian, Black and Caribbean Participants of the UK Biobank
Source: Front Genet. 2022 Mar 31;13:845498. doi: 10.3389/fgene.2022.845498 (PMC9010053; doi:10.3389/fgene.2022.845498)
Supplement: Supplementary file 1 [file DataSheet1.docx]

Supplementary Material

**Supplementary Table 1. SNPs used in the 12-SNP score.** **APOE* weights are isoform dependent as described in Talmud *et al*(Talmud et al. 2013). GLGC = Global Lipids Genetics Consortium; GRCh37 = Genome Reference Consortium Human Build 37; SNP = single nucleotide polymorphism.

| **SNP** | **Gene** | **Chromosome number** | **Start position (GRCh37)** | **Common allele** | **Alternate allele** | **GLGC weight for score calculation** | **UKB allele frequencies** | | | **P-values for t-test of allele frequencies** | |
| --- | --- | --- | --- | --- | --- | --- | --- | --- | --- | --- | --- |
|  |  |  |  |  |  |  | **White** | **Black & Caribbean** | **South Asian** | **White-Black & Caribbean** | **White-South Asian** |
| **rs2479409** | *PCSK9* | 1 | 55504650 | G | A | 0.052 | 0.6516 | 0.7484 | 0.7481 | < 2.2x10^-16^ | < 2.2x10^-16^ |
| **rs629301** | *CELSR2* | 1 | 109818306 | G | T | -0.15 | 0.7777 | 0.6297 | 0.7362 | < 2.2x10^-16^ | < 2.2x10^-16^ |
| **rs1367117** | *APOB* | 2 | 21263900 | G | A | -0.1 | 0.3388 | 0.0856 | 0.1584 | < 2.2x10^-16^ | < 2.2x10^-16^ |
| **rs4299376** | *ABCG8* | 2 | 44072576 | G | T | 0.071 | 0.6766 | 0.8469 | 0.7413 | < 2.2x10^-16^ | < 2.2x10^-16^ |
| **rs1564348** | *SLC22A1* | 6 | 160578860 | T | C | 0.014 | 0.1705 | 0.1078 | 0.1486 | < 2.2x10^-16^ | 1.1x10^-12^ |
| **rs1800562** | *HFE* | 6 | 26093141 | G | A | 0.057 | 0.0777 | 0.0055 | 0.0022 | < 2.2x10^-16^ | < 2.2x10^-16^ |
| **rs3757354** | *MYLIP* | 6 | 16127407 | C | T | 0.037 | 0.2022 | 0.3700 | 0.3020 | < 2.2x10^-16^ | < 2.2x10^-16^ |
| **rs11220462** | *ST3GAL4* | 11 | 126243952 | G | A | -0.050 | 0.1325 | 0.0258 | 0.1373 | < 2.2x10^-16^ | 0.1 |
| **rs8017377** | *NYNRIN* | 14 | 24883887 | G | A | -0.029 | 0.4748 | 0.1338 | 0.3938 | < 2.2x10^-16^ | < 2.2x10^-16^ |
| **rs6511720** | *LDLR* | 19 | 11202306 | G | T | 0.18 | 0.1189 | 0.1423 | 0.0779 | 2.8x10^-15^ | < 2.2x10^-16^ |
| **rs429358** | *APOE** | 19 | 45411941 | T | C |  | 0.1563 | 0.2298 | 0.0967 | < 2.2x10^-16^ | < 2.2x10^-16^ |
| **rs7412** | *APOE** | 19 | 45412079 | C | T |  | 0.0810 | 0.1130 | 0.0442 | < 2.2x10^-16^ | < 2.2x10^-16^ |
| **ε2ε2 (rs429358: TT / rs7412: TT)** | *APOE** | 19 |  |  |  | -0.9 |  |  |  |  |  |
| **ε2ε3 (rs429358: TT / rs7412: TC)** | *APOE** | 19 |  |  |  | -0.4 |  |  |  |  |  |
| **ε2ε4 (rs429358: TC / rs7412: TC)** | *APOE** | 19 |  |  |  | -0.2 |  |  |  |  |  |
| **ε3ε3 (rs429358: TT / rs7412: CC)** | *APOE** | 19 |  |  |  | 0 |  |  |  |  |  |
| **ε3ε4 (rs429358: TC / rs7412: CC)** | *APOE** | 19 |  |  |  | 0.1 |  |  |  |  |  |
| **ε4ε4 (rs429358: CC / rs7412: CC)** | *APOE** | 19 |  |  |  | 0.2 |  |  |  |  |  |

**Supplementary Table 2. Relationship between the 12-SNP score and LDL-C concentrations in principal component analysis (PCA)-verified White European, Black, and South Asian ancestries.** R^2^ and beta coefficient for LDL-C, the area under the curve (AUC) and the odds ratio (OR) for hypercholesterolaemia (LDL-C >4.9 mmol/L) using European ancestry deciles and ancestry-specific 12-SNP score decile cut-off values in Black and South Asian ancestries. The results presented here are using the test data. The training data was used to obtain the 12-SNP score decile cut-off values for each ethnic group studied. Low PH (polygenic hypercholesterolaemia) refers to deciles 1-3, intermediate PH are for deciles 4-5, and high PH include deciles 6-10. * denotes the interaction term in the regression analysis. AUC = area under the curve; CI = confidence interval; LDL-C = low-density lipoprotein cholesterol; OR = odds ratio; PH = polygenic hypercholesterolaemia; SNP = single nucleotide polymorphism.

| Outcomes and model predictors | **White ancestry** | | | **Black ancestry** | | | **South Asian ancestry** | | |
| --- | --- | --- | --- | --- | --- | --- | --- | --- | --- |
| **Outcome: LDL-C** | **R^2^** | **Lower 95% CI** | **Upper 95% CI** | **R^2^** | **Lower 95% CI** | **Upper 95% CI** | **R^2^** | **Lower 95% CI** | **Upper 95% CI** |
| Model predictors: **12-SNP score** | 0.068 | 0.066 | 0.07 | 0.083 | 0.07 | 0.096 | 0.033 | 0.025 | 0.041 |
| Model predictors: **12-SNP score + age * sex** | 0.11 | 0.108 | 0.112 | 0.11 | 0.096 | 0.124 | 0.049 | 0.039 | 0.059 |
| **Outcome: LDL-C** | **Beta** | **Lower 95% CI** | **Upper 95% CI** | **Beta** | **Lower 95% CI** | **Upper 95% CI** | **Beta** | **Lower 95% CI** | **Upper 95% CI** |
| Model predictors: **12-SNP score** | 0.261 | 0.247 | 0.274 | 0.288 | 0.207 | 0.369 | 0.182 | 0.074 | 0.290 |
| **Outcome: LDL-C >4.9 mmol/L** | **OR** | **Lower 95% CI** | **Upper 95% CI** | **OR** | **Lower 95% CI** | **Upper 95% CI** | **OR** | **Lower 95% CI** | **Upper 95% CI** |
| Model predictors: **12-SNP score** | 11.26 | 10.48 | 12.09 | 10.38 | 6.13 | 17.87 | 7.54 | 4.24 | 13.61 |
| **Outcome: CHD prevalence** | **OR** | **Lower 95% CI** | **Upper 95% CI** | **OR** | **Lower 95% CI** | **Upper 95% CI** | **OR** | **Lower 95% CI** | **Upper 95% CI** |
| Model predictors: **12-SNP score** | 1.82 | 1.66 | 2.01 | 1.35 | 0.63 | 3.03 | 1.13 | 0.69 | 1.90 |
| **Outcome: CHD incidence** | **OR** | **Lower 95% CI** | **Upper 95% CI** | **OR** | **Lower 95% CI** | **Upper 95% CI** | **OR** | **Lower 95% CI** | **Upper 95% CI** |
| Model predictors: **12-SNP score** | 1.36 | 1.25 | 1.49 | 1.72 | 0.93 | 3.26 | 1.87 | 1.12 | 3.16 |
| **Outcome: LDL-C** | **R2** | **Lower 95% CI** | **Upper 95% CI** | **R2** | **Lower 95% CI** | **Upper 95% CI** | **R2** | **Lower 95% CI** | **Upper 95% CI** |
| Model predictors: **ancestry-specific deciles** | 0.059 | 0.057 | 0.061 | 0.072 | 0.06 | 0.084 | 0.027 | 0.019 | 0.035 |
| Model predictors: **WB deciles** | 0.059 | 0.057 | 0.061 | 0.059 | 0.048 | 0.07 | 0.028 | 0.02 | 0.036 |
| **Outcome:** **LDL-C >4.9 mmol/L** | **AUC** | **Lower 95% CI** | **Upper 95% CI** | **AUC** | **Lower 95% CI** | **Upper 95% CI** | **AUC** | **Lower 95% CI** | **Upper 95% CI** |
| Model predictors: **ancestry-specific deciles** | 0.63 | 0.63 | 0.64 | 0.64 | 0.61 | 0.67 | 0.60 | 0.57 | 0.63 |
| Model predictors: **WB deciles** | 0.63 | 0.63 | 0.64 | 0.64 | 0.61 | 0.68 | 0.60 | 0.57 | 0.63 |
| **Outcome:** **LDL-C >4.9 mmol/L** | **OR** | **Lower 95% CI** | **Upper 95% CI** | **OR** | **Lower 95% CI** | **Upper 95% CI** | **OR** | **Lower 95% CI** | **Upper 95% CI** |
| Model predictors: **ancestry-specific deciles** | 1.18 | 1.17 | 1.19 | 1.20 | 1.15 | 1.25 | 1.13 | 1.10 | 1.18 |
| Model predictors: **WB deciles** | 1.18 | 1.17 | 1.19 | 1.21 | 1.16 | 1.26 | 1.14 | 1.10 | 1.19 |
| **Outcome:** **LDL-C >4.9 mmol/L** | **Low PH (OR (95% CI)** | **Intermediate PH (OR (95% CI)** | **High PH (OR (95% CI)** | **Low PH (OR (95% CI)** | **Intermediate PH (OR (95% CI)** | **High PH (OR (95% CI)** | **Low PH (OR (95% CI)** | **Intermediate PH (OR (95% CI)** | **High PH (OR (95% CI)** |
| Model predictors: **ancestry-specific deciles** | 0.53 (0.51-0.56) | 0 (reference) | 1.60 (1.54-1.66) | 0.50 (0.34-0.73) | 0 (reference) | 1.63 (1.23-2.17) | 0.53 (0.38-0.73) | 0 (reference) | 1.31 (1.03-1.69) |
| Model predictors: **WB deciles** | 0.53 (0.51-0.56) | 0 (reference) | 1.60 (1.54-1.66) | 0.61 (0.47-0.81) | 0 (reference) | 1.62 (1.22-2.17) | 0.59 (0.45-0.78) | 0 (reference) | 1.36 (1.09-1.72) |

**Supplementary Table 3. Relationship between the 12-SNP score and LDL-C concentrations: R^2^ for LDL-C, the area under the curve (AUC) and the odds ratio (OR) for hypercholesterolaemia (LDL-C >4.9 mmol/L) using WB and ethnicity-specific 12-SNP score decile cut-off values.** The results presented here are using the test data. The training data was used to obtain the 12-SNP score decile cut-off values for each ethnic group studied. Low PH (polygenic hypercholesterolaemia) refers to deciles 1-3, intermediate PH are for deciles 4-5, and high PH include deciles 6-10. * denotes the interaction term in the regression analysis. AUC = area under the curve; CI = confidence interval; LDL-C = low-density lipoprotein cholesterol; OR = odds ratio; PH = polygenic hypercholesterolaemia; SNP = single nucleotide polymorphism; WB = White British.

| Outcomes and model predictors | **White ethnicity** | | | | | **Black/Caribbean ethnicities** | | | | | | **South Asian ethnicity** | | | | | |
| --- | --- | --- | --- | --- | --- | --- | --- | --- | --- | --- | --- | --- | --- | --- | --- | --- | --- |
| **Outcome: LDL-C** | **R^2^** | **Lower 95% CI** | | **Upper 95% CI** | | **R^2^** | | **Lower 95% CI** | | **Upper 95% CI** | | **R^2^** | | **Lower 95% CI** | | **Upper 95% CI** | |
| Model predictors: **12-SNP score** | 0.067 | 0.065 | | 0.069 | | 0.080 | | 0.063 | | 0.097 | | 0.027 | | 0.016 | | 0.038 | |
| Model predictors: **12-SNP score + age * sex** | 0.108 | 0.105 | | 0.111 | | 0.105 | | 0.086 | | 0.124 | | 0.049 | | 0.035 | | 0.063 | |
| **Outcome: LDL-C** | **Beta** | | **Lower 95% CI** | | **Upper 95% CI** | | **Beta** | | **Lower 95% CI** | | **Upper 95% CI** | | **Beta** | | **Lower 95% CI** | | **Upper 95% CI** |
| Model predictors: **12-SNP score** | 0.258 | | 0.241 | | 0.275 | | 0.282 | | 0.169 | | 0.395 | | 0.163 | | 0.012 | | 0.315 |
| **Outcome: LDL-C >4.9 mmol/L** | **OR** | | **Lower 95% CI** | | **Upper 95% CI** | | **OR** | | **Lower 95% CI** | | **Upper 95% CI** | | **OR** | | **Lower 95% CI** | | **Upper 95% CI** |
| Model predictors: **12-SNP score** | 11.01 | | 10.08 | | 12.04 | | 10.54 | | 5.29 | | 21.67 | | 6.64 | | 2.98 | | 15.22 |
| **Outcome: CHD prevalence** | **OR** | | **Lower 95% CI** | | **Upper 95% CI** | | **OR** | | **Lower 95% CI** | | **Upper 95% CI** | | **OR** | | **Lower 95% CI** | | **Upper 95% CI** |
| Model predictors: **12-SNP score** | 1.76 | | 1.56 | | 1.99 | | 2.26 | | 0.78 | | 7.22 | | 1.24 | | 0.62 | | 2.53 |
| **Outcome: CHD incidence** | **OR** | | **Lower 95% CI** | | **Upper 95% CI** | | **OR** | | **Lower 95% CI** | | **Upper 95% CI** | | **OR** | | **Lower 95% CI** | | **Upper 95% CI** |
| Model predictors: **12-SNP score** | 1.25 | | 1.13 | | 1.39 | | 1.09 | | 0.48 | | 2.58 | | 1.98 | | 0.95 | | 4.25 |
| **Outcome: LDL-C** | **R2** | | **Lower 95% CI** | | **Upper 95% CI** | | **R2** | | **Lower 95% CI** | | **Upper 95% CI** | | **R2** | | **Lower 95% CI** | | **Upper 95% CI** |
| Model predictors: **ethnicity-specific deciles** | 0.058 | | 0.056 | | 0.060 | | 0.063 | | 0.047 | | 0.079 | | 0.022 | | 0.012 | | 0.032 |
| Model predictors: **WB deciles** | 0.058 | | 0.056 | | 0.060 | | 0.074 | | 0.057 | | 0.091 | | 0.021 | | 0.012 | | 0.030 |
| **Outcome:** **LDL-C >4.9 mmol/L** | **AUC** | | **Lower 95% CI** | | **Upper 95% CI** | | **AUC** | | **Lower 95% CI** | | **Upper 95% CI** | | **AUC** | | **Lower 95% CI** | | **Upper 95% CI** |
| Model predictors: **ethnicity-specific deciles** | 0.63 | | 0.63 | | 0.64 | | 0.65 | | 0.61 | | 0.69 | | 0.59 | | 0.55 | | 0.63 |
| Model predictors: **WB deciles** | 0.63 | | 0.63 | | 0.64 | | 0.65 | | 0.61 | | 0.69 | | 0.59 | | 0.55 | | 0.63 |
| **Outcome:** **LDL-C >4.9 mmol/L** | **OR** | | **Lower 95% CI** | | **Upper 95% CI** | | **OR** | | **Lower 95% CI** | | **Upper 95% CI** | | **OR** | | **Lower 95% CI** | | **Upper 95% CI** |
| Model predictors: **ethnicity-specific deciles** | 1.18 | | 1.17 | | 1.19 | | 1.21 | | 1.15 | | 1.28 | | 1.12 | | 1.07 | | 1.18 |
| Model predictors: **WB deciles** | 1.18 | | 1.17 | | 1.19 | | 1.24 | | 1.17 | | 1.31 | | 1.14 | | 1.08 | | 1.21 |
| **Outcome:** **LDL-C >4.9 mmol/L** | **Low PH (OR (95% CI)** | | **Intermediate PH (OR (95% CI)** | | **High PH (OR (95% CI)** | | **Low PH (OR (95% CI)** | | **Intermediate PH (OR (95% CI)** | | **High PH (OR (95% CI)** | | **Low PH (OR (95% CI)** | | **Intermediate PH (OR (95% CI)** | | **High PH (OR (95% CI)** |
| Model predictors: **ethnicity-specific deciles** | 0.54 (0.51-0.57) | | 0 (reference) | | 1.60 (1.53-1.67) | | 0.40 (0.24-0.68) | | 0 (reference) | | 1.56 (1.08-2.31) | | 0.75 (0.48-1.16) | | 0 (reference) | | 1.50 (1.05-2.20) |
| Model predictors: **WB deciles** | 0.54 (0.51-0.57) | | 0 (reference) | | 1.60 (1.53-1.67) | | 0.56 (0.39-0.80) | | 0 (reference) | | 1.56 (1.07-2.28) | | 0.84 (0.57-1.23) | | 0 (reference) | | 1.70 (1.22-2.40) |

**Supplementary Table 4. Outcome data when applying the WB 12-SNP score deciles to WB, BC, and SA ethnicities in the test data.** The results presented here are using the test data. The training data was used to obtain the 12-SNP score decile cut-off values for each ethnic group studied. BC = Black and Caribbean; LDL-C = low-density lipoprotein cholesterol; SD = standard deviation; SA = South Asian; SNP = single nucleotide polymorphism; WB = White British.

| **Self-reported ethnicity** | **12-SNP score decile** | **Number of individuals** | **Mean 12-SNP score** | **SD 12-SNP score** | **Mean LDL-C (mmol/L)** | **SD LDL-C (mmol/L)** | **Number of individuals with LDL-C >4.9 mmol/L** | **Percent of individuals with LDL-C >4.9 mmol/L** |
| --- | --- | --- | --- | --- | --- | --- | --- | --- |
| White | 1 | 17731 | 0.42 | 0.15 | 3.27 | 0.78 | 505 | 2.8 |
| White | 2 | 17846 | 0.66 | 0.04 | 3.49 | 0.77 | 746 | 4.2 |
| White | 3 | 17457 | 0.77 | 0.03 | 3.61 | 0.79 | 1018 | 5.8 |
| White | 4 | 17915 | 0.85 | 0.02 | 3.69 | 0.82 | 1320 | 7.4 |
| White | 5 | 17707 | 0.91 | 0.02 | 3.74 | 0.82 | 1422 | 8 |
| White | 6 | 17371 | 0.96 | 0.01 | 3.8 | 0.83 | 1633 | 9.4 |
| White | 7 | 17395 | 1.01 | 0.01 | 3.83 | 0.85 | 1758 | 10.1 |
| White | 8 | 17551 | 1.06 | 0.02 | 3.89 | 0.84 | 1983 | 11.3 |
| White | 9 | 17920 | 1.12 | 0.02 | 3.94 | 0.86 | 2230 | 12.4 |
| White | 10 | 17150 | 1.22 | 0.06 | 4.03 | 0.88 | 2682 | 15.6 |
| Black & Caribbean | 1 | 895 | 0.37 | 0.19 | 3.06 | 0.82 | 20 | 2.2 |
| Black & Caribbean | 2 | 681 | 0.66 | 0.04 | 3.4 | 0.84 | 31 | 4.6 |
| Black & Caribbean | 3 | 508 | 0.77 | 0.02 | 3.43 | 0.91 | 32 | 6.3 |
| Black & Caribbean | 4 | 452 | 0.85 | 0.02 | 3.49 | 0.78 | 20 | 4.4 |
| Black & Caribbean | 5 | 300 | 0.91 | 0.01 | 3.71 | 0.92 | 32 | 10.7 |
| Black & Caribbean | 6 | 254 | 0.96 | 0.01 | 3.71 | 0.97 | 20 | 7.9 |
| Black & Caribbean | 7 | 162 | 1 | 0.01 | 3.83 | 0.96 | 14 | 8.6 |
| Black & Caribbean | 8 | 126 | 1.05 | 0.01 | 3.65 | 1.07 | 16 | 12.7 |
| Black & Caribbean | 9 | 87 | 1.12 | 0.02 | 3.81 | 0.97 | 14 | 16.1 |
| Black & Caribbean | 10 | 27 | 1.22 | 0.07 | 3.96 | 0.79 | 4 | 14.8 |
| South Asian | 1 | 301 | 0.45 | 0.12 | 3.27 | 0.82 | 11 | 3.7 |
| South Asian | 2 | 424 | 0.67 | 0.04 | 3.45 | 0.79 | 20 | 4.7 |
| South Asian | 3 | 482 | 0.77 | 0.02 | 3.52 | 0.83 | 25 | 5.2 |
| South Asian | 4 | 516 | 0.85 | 0.02 | 3.55 | 0.81 | 27 | 5.2 |
| South Asian | 5 | 447 | 0.91 | 0.01 | 3.61 | 0.83 | 26 | 5.8 |
| South Asian | 6 | 427 | 0.96 | 0.01 | 3.62 | 0.9 | 31 | 7.3 |
| South Asian | 7 | 329 | 1.01 | 0.01 | 3.75 | 0.97 | 36 | 10.9 |
| South Asian | 8 | 252 | 1.05 | 0.02 | 3.65 | 0.91 | 22 | 8.7 |
| South Asian | 9 | 184 | 1.12 | 0.02 | 3.79 | 0.95 | 17 | 9.2 |
| South Asian | 10 | 94 | 1.21 | 0.05 | 3.86 | 0.85 | 10 | 10.6 |
